# Assessing the Time for Living and Caring (TLC) Study: Mixed-Methods Feasibility Study of a Web-Based Caregiver Intervention to Improve Respite

**DOI:** 10.2196/71792

**Published:** 2025-08-18

**Authors:** Amber D Thompson, Alexandra L Terrill, Michael Caserta, Bob Wong, Eli Iacob, Catharine Sparks, Louisa Stark, Rebecca L Utz

**Affiliations:** 1 College of Social & Behavioral Science, University of Utah, 260 S Central Campus Dr, Salt Lake City, UT, 84112, United States, 1 801-581-6153; 2 College of Health, University of Utah, Salt Lake City, UT, United States; 3 College of Nursing, University of Utah, Salt Lake City, UT, United States; 4 School of Medicine, University of Utah, Salt Lake City, UT, United States

**Keywords:** respite care, internet-based intervention, feasibility studies

## Abstract

**Background:**

Interventions that are self-administered and delivered online are increasingly being seen as a flexible way to support family caregivers. Intervention research should prioritize the measurement of feasibility throughout all of the stages of intervention development and evaluation to provide the essential feedback loop needed for the iterative development and refinement process.

**Objective:**

We describe the methodology and data used to assess the feasibility, usability, and acceptability of the Time for Living and Caring (TLC) intervention, a technology-delivered intervention (app) for dementia caregivers to improve respite time use.

**Methods:**

The feasibility analysis is theoretically guided by a multidimensional definition of feasibility and uses a mixed-methods research design. Stakeholder feedback collected via focus groups during intervention development (n=15), self-reported surveys from participants enrolled in the pilot trial of the intervention (n=163), surveys of a nationwide sample of respite providers (n=57), and end-user statistics, captured passively by Google Analytics from those using the app, were used in the feasibility analysis of the TLC intervention.

**Results:**

The TLC study used an appropriate design and data collection procedures, along with acceptable recruitment capability. Out of 5 intervention features, 4 received favorable ratings (range of 82%‐99%) by intervention participants and respite providers, which, when combined with open-ended recommendations for improvements, indicate a high degree of usability. Acceptability was measured through appraisal of the intervention experience (135/159, 85% positive), potential future use (127/163, 78%), willingness to recommend (148/163, 91%), and perceived benefit (135/163, 83%).

**Conclusions:**

Taken together, the data suggest that the TLC app is a promising intervention that could be implemented as an on-demand resource for respite-using caregivers, irrespective of where they are located or when they choose to access it. Additionally, this paper provides a blueprint for systematically evaluating multiple dimensions of feasibility, using various forms of mixed-methods data collected during intervention development and pilot testing of an intervention, which should help streamline the eventual implementation of effective interventions in real-world settings.

## Introduction

### Family Caregivers and Need for Interventions

As the population demand for family caregivers increases [1] and as there is greater public awareness of both the value that caregivers provide [2] and the challenges that they face [3,4], there has been a call for the development and implementation of caregiver support programs [5]. As a result, and not surprisingly, there has been a proliferation of new evidence-based and research-informed caregiver interventions in recent years [6-9].

Respite—defined as a planned break or time away from caregiving [10]—is among the most needed and requested forms of caregiver support [11]; yet, few caregiver interventions address respite use, likely reflecting a lack of understanding of why there are low rates of respite uptake among family caregivers [1] or the mechanisms underlying the negative and mixed results regarding the effectiveness of respite overall [12]. Nevertheless, empirical research showing how caregivers used their respite time and how respite time use affected respite satisfaction and overall well-being [13,14] paved the way for the development of a novel respite-focused intervention called the Time for Living and Caring (TLC). TLC provides caregivers with coaching and educational resources to schedule and plan their respite time use. Based on the well-researched “Selective Optimization with Compensation” model [15], TLC uses weekly goal-setting and goal-review exercises to help caregivers schedule and plan respite and increase self-awareness about their need for respite. It was hypothesized that with better respite time use, caregivers would have better overall well-being and less caregiving-related stress [16].

TLC, like many of the other newer caregiver support interventions [17,18], was developed to be self-administered and delivered via an interactive website or “app”—with the rationale that online delivery is a flexible way to support caregivers who are often time-limited and place-bound, given the demands of providing round-the-clock care to someone in the home [19]. However, many have questioned the appropriateness of using web-based technologies with an older caregiving population (the average age of caregivers in the United States is 49, with 54% 50 y and older) [1]. Older adults are thought to be less interested, able, or willing to use web-based technologies for support and have, on average, less access to the internet and internet-capable devices and lower computer proficiency to access online resources [20], potentially threatening the feasibility of the TLC intervention and reducing its potential to support family caregivers.

### Intervention Development and Evaluation Studies

Intervention research starts with an intervention idea, which is often guided by a theoretical model or previous stage-01 empirical research findings. Once developed, interventions should be pilot-tested, followed by more formal testing and evaluation in various controlled and real-world settings. The final stage is implementation into existing clinical or community-based settings (see National Institute of Health stage model) [21,22]. There are a variety of methodological approaches and recommended actions at each stage [23]. For example, current best practices recommend using community-engaged partnerships or stakeholders to help the researchers co-develop the features of the intervention [24], and using randomized controlled trials during the formal evaluation and testing stages. Although there is a clear preference for evidence-informed practices, there exists little consensus on the specific strategies that should be used, the data that should be collected, or the metrics that should be analyzed at each stage of intervention development to maximize the intervention’s effectiveness and eventual implementation [25].

Rarely is there a clear delineation of when the development stage ends and when pilot testing and formal evaluation should begin. This may be due, in part, to each stage of intervention research emphasizing the need to formally assess feasibility. *Feasibility* is the broad concept used to describe the possibility, readiness, or likely success of an intervention. Gadke et al [26] provide a comprehensive and multidimensional definition of feasibility, which includes 10 distinct considerations related to (1) design procedures, (2) data collection procedures, (3) recruitment capability, (4) practicality, (5) adaptability, (6) social validity, (7) generalizability, (8) integration into existing systems, (9) implementation, and (10) effectiveness (refer to Table 1 for more information about each dimension). Gadke et al’s [26] multi-dimensional definition of feasibility has been used as a framework to guide intervention development and implementation [27-29].

**Table 1.** Dimensions of feasibility. Adapted from Gadke et al [26].

Dimension	Conceptual description	Metric or research methodology used to assess
Design procedures	Is the research design appropriate and sensitive to evaluating change?	Randomized controlled trial (RCT), with a modified waitlist control design; See Figure 1. TLC study design
Data collection procedures	Are data collection procedures and outcome measures appropriate and sensitive to change?	Participant self-report surveys (n=6), spaced every 4 weeks over a 20-week time frame
Recruitment capability	Can participants who will benefit from and who will implement the intervention be identified and successfully recruited for study?	See Figure 2 and Table 2
Practicality	Can the intervention be implemented with available resources, time, training, and materials?	See Table 3
Adaptability	Is there sufficient flexibility built into the intervention procedures to accommodate diverse needs?	See Table 3
Social validity	Does the intervention have social significance or relevance? (traditionally conceptualized as acceptability)	See Table 4
Generalizability	Does the intervention generalize to non-research settings, over time, or with diverse samples?	See Table 4
Integration into existing systems	To what extent does the intervention align with the infrastructure of the relevant practice setting or system?	See Table 4 and Table 5
Implementation	Can providers implement the intervention with fidelity?	Online “app” standardizes delivery and fidelity.
Effectiveness	Is there any preliminary evidence of the intervention effect in bringing about positive change?	See Table 6

**Table 2.** Practicality and adaptability of TLC intervention (n=163^a^).

Indicator/variable	Mean (SD) or percent	Min	Max
Owned internet capable device	96%	—	—
Had internet access	100%	—	—
Preferred device to access intervention			
Desktop computer	23%		
Laptop computer	36%		
Tablet, such as an iPad	13%		
Smart phone	28%		
Other	<1%		
Computer proficiency (scale range 12‐60)	56.12 (5.14)	35	60
Number of sessions	20.44 (13.84)	0	67
Average duration per session (in *seconds*)	506 (313)	72	1996
Total Time (in *hours*) per enrollee-recruitment	12.6	-	-
Cost per enrollee-recruitment	$205	-	-

a All data come from the TLC sample of dementia caregivers and include Google Analytics, self-reported survey responses by caregivers, and cost analysis.

**Table 3.** Feedback about the TLC^a^ app from caregivers (n=132) and from providers (n=57).

	Dementia caregivers	Respite providers
Usefulness or importance of specific TLC features^b^		
Introductory video	86%	97%
Calendar	90%	90%
Coaching (goal setting/review)	88%	95%
Dashboard	82%	85%
Resources	67%	98%
Future use and delivery of TLC intervention		
Would recommend app to others	91%	89%
Would use app in future if available	78%	—
App could be used by all caregivers (not just dementia caregivers)	—	75%

a TLC: Time for Living and Caring.

b Caregivers were asked about the usefulness of each feature; providers were asked about the importance of each intervention feature.

**Table 4.** Exemplars of why participants would or would not use a TLC smartphone app.

YES—I would use	NO—I would not use
*I rarely use my laptop anymore, just my cell phone. That way, I can get on and do it any time, anywhere*.	*I don’t like using the small screen for calendaring*.
*I ALWAYS have my phone with me*.	*I don’t like using app’s on my phone. I try not to have it glued to me. I like to use different devices and phone is not one of them. I spend the majority of my day at a computer working so picking up a phone after that is not at the top of my list*.
*Simpler, easier to incorporate into my existing life. Being a caregiver--for me--means I don’t have as much time to spend on the computer*.	
*It would be much better as an app. Sometimes having to go the extra work of only being able to access the website from my desktop actually added a little more stress*.	*My poor old arthritic fingers have to type too much on a tiny iPhone 8+ keyboard already. So much texting. Everyone wants to text! Sometimes a pain. And after many hours, some strain on the eyes. My notebook easier - 13” screen. Bigger keyboard. And I do not want one more app on my phone. But that’s just me*...
*it would be convenient to access when I’m at the doctor’s or am waiting in line. I spend a lot of down time at the doctors*.	
*Easier for people who don’t have or do well on tablets and computers*.	*That’s actually a “maybe” depending on how well the app works. For me it was useful to sit down at the computer once a week and do the planning.*

Traditionally, a feasibility study is conducted prior to an outcomes-focused pilot study and before any formal full-scale evaluation effort to test an intervention’s effectiveness. However, the terms pilot study and feasibility study are often used interchangeably, reinforcing the importance of systematically assessing feasibility during the early stages of intervention development. Pilot studies are used to “try out” an intervention and to develop research procedures that may provide clarity and input on the mechanisms underlying the intervention’s effectiveness in general and within specific and unique contexts [30]. This is what Chen [31] calls “viability validity”—evidence that an intervention is practical, affordable, suitable, valuable, and helpful to people using it in the real world. Conducting systematic and rigorous evaluations of an intervention’s feasibility can help to avoid wasting time, money, and resources and ultimately lead to an intervention that is feasible, acceptable, or effective for the intended purpose and population [23,25,32]. Conducting a systematic and comprehensive feasibility analysis provides the essential feedback loop needed for the iterative development and refinement process as interventions are developed, tested, and eventually implemented [32,33].

### The Present Study

The objective of this paper is to comprehensively assess the feasibility of the TLC respite-focused caregiver intervention (for an Assessing Feasibility of an Online Caregiver Intervention to Improve Respite: The Time for Living and Caring (TLC) Study of TLC intervention outcomes, see Iacob et al [34]). Our analysis is theoretically guided by the multidimensional definition of feasibility offered by Gadke et al [26] and uses multiple sources of data from the National Institute on Aging funded pilot study of the TLC intervention (NIA R01–AG061946). Results provide specific evidence of whether TLC, an online web-based intervention, is a potentially promising new intervention model to support family caregivers. Additionally, a review of our evaluation methods and metrics provides a blueprint for how other researchers can assess feasibility during the early stages of intervention development and pilot testing. Systematic and comprehensive evaluation of feasibility—at all stages of intervention research—is imperative if the goal is to implement the most effective and potent programs to support and address the needs of the target or intended population [21]—in this case, the 1 in 5 American adults (21.3% or 53 million) who provide an estimated $600 billion worth of unpaid care and support to an adult or child with special needs in our communities [1,2]. Therefore, the aims of this study are twofold: (1) to assess the feasibility of the TLC respite-focused caregiver intervention and (2) to provide an example and process of how to assess feasibility during the early stages of intervention development, refinement, and pilot testing.

## Methods

### Data

Data come from the TLC research study, a pilot study funded by the National Institute on Aging (R01—AG061946) with specific aims to refine and evaluate the TLC online respite-focused intervention (“app”) among a sample of dementia caregivers. In total, 4 data sources are used to conduct our feasibility analysis: (1) stakeholder feedback collected via focus groups conducted during the intervention development stage (n=15 community advisory board members), (2) self-reported surveys from participants enrolled in the pilot trial of the intervention (n=163), (3) end-user statistics captured passively by Google Analytics from those using the app, and (4) surveys of a nationwide sample of respite providers (n=57) (ie, health or social care professionals who are supporting family caregivers with service delivery or advocacy). More information about the TLC study, including results on the efficacy of TLC to improve caregiver outcomes [34] and access to an archive of all de-identified data and study protocols, can be found on the HIVE archive at the University of Utah [35]. A free, publicly available version of the TLC intervention can be found online [36] (see Multimedia Appendix 1for intervention screenshots).

#### Ethical Considerations

All study procedures were approved by the Institutional Review Board at the University of Utah (# 00120589) and were pre-registered via Clinical Trials (NCT03689179) [37]. Informed consent was obtained from participants after the nature and possible consequences of the studies were explained. To protect participants’ privacy and confidentiality, no identifying details have been included.

### Measures

The primary outcome for this analysis is feasibility. Using a multidimensional framework for feasibility, Table 1 describes the 10 dimensions of feasibility [26], as well as a description of the specific types of data or metrics we used to assess each dimension: (1) design procedures, (2) data collection procedures, (3) recruitment capability, (4) practicality, (5) adaptability, (6) social validity, (7) generalizability, (8) integration into existing systems, (9) implementation, and (10) effectiveness.

**Figure 1.**
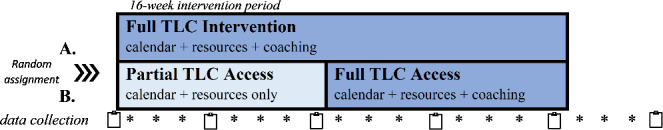
TLC (Time for Living and Caring) study design.

### Analytic Strategy

Following recommendations by Aschbrenner et al [38], we used a mixed-methods study design to conduct this feasibility analysis. We first identified which data could be used to assess each of the 10 dimensions of feasibility (eg, rating by study participants of their overall experience with the intervention versus open-ended comments about what they liked most or least). Next, we aligned quantitative and qualitative data sources for the feasibility domain(s) selected (eg, specifying benchmarks and identifying the most relevant participants and sources for the selected feasibility domain(s)). A third strategy was to determine the timing and methodology needed for each assessment (ie, focus groups, study surveys, and provider surveys collected throughout the intervention development and pilot testing). Next, we planned integrative analyses using joint displays to understand feasibility. For example, using a traditional CONSORT diagram, used to describe sample representativeness in a randomized controlled trial study design, along with a descriptive table that compares the study sample to national caregiver population characteristics, allows for a comprehensive and nuanced understanding of the domain of recruitment capability. The last step involved interpreting the quantitative, qualitative, and mixed-methods feasibility results to draw conclusions and meta-inferences about the intervention (eg, the implications and possibilities for intervention refinement that may optimize intervention effectiveness).

## Results

### Overview

This section provides a description and discussion of the specific metrics, research methodologies, and analyses used to assess the TLC intervention on each of the 10 dimensions of feasibility.

### Study Design

The larger TLC study adopted a comprehensive set of mixed-methods research activities and a randomized controlled trial to conduct the formal pilot testing of the TLC intervention (ie, Stage 1A and Stage 1B of the National Institute on Aging stage model for behavioral interventions [21]). This resulted in 4 distinct sources of data that could be used for the feasibility analysis: (1) qualitative feedback from a 15-member community advisory board who worked closely with the research team, software developers, and creative specialists (writers, media producers, graphic designers) to redevelop and refine the conceptual features of TLC intervention into a self-administered web-based intervention platform (app) that reflected the diverse need and experiences of caregivers, (2) a fully-powered pilot sample of dementia caregivers who had access to the TLC intervention for 16 weeks and who completed self-report questionnaires every week for 20 weeks (n=163), (3) end-user statistics describing engagement with the web-based intervention platform passively captured by Google Analytics for all intervention participants (n=163), and (4) a nationwide sample of respite providers (n=57), who offered an additional layer of quantitative and qualitative data regarding the feasibility and usability of the intervention, with a particular focus on its potential implementation within existing caregiver support services and networks.

As shown in Figure 1, the TLC participant sample used a modified waitlist control design, where participants were assigned to either Group A (the full treatment group, where they received all components of the interventions for the full 16 wk intervention period) or Group B (the partial or delayed access group, where they received the full intervention after an initial 8 wk period during which they only had partial access to the intervention). This randomized controlled trial study design allows each participant to serve as their own control (ie, baseline pre-intervention compared to any other survey), while also preserving the powerful inter-group comparisons that come with randomized controlled designs.

### Data Collection

To ensure sensitivity to change, the TLC study used frequent and consistent measurements of outcomes via monthly surveys (baseline, weeks 4, 8, 12, 16, and 20). During enrollment, participants provided information to assess eligibility and provided an email that could be used as a contact throughout the study to send survey links and reminders. Upon enrollment, participants were emailed a baseline survey that included characteristic measures (eg, sociodemographic, caregiving situation, and computer proficiency), pre and post measures (eg, positive affect, desire to institutionalize, and respite characteristics), and repeated measures (eg, depression, anxiety, and burden). We used reliable instruments (eg, the PROMIS Depression short-form questionnaire for adults, an 8-item additive scale that standardizes the distribution of depression-related symptoms on a population distribution) [39], implemented standardized protocols including automated text and targeted email reminders, and collected data across multiple time points. Specifically, participants were emailed a repeated measures survey every 4 weeks after completion of the baseline survey up to week 16. At week 20 (conclusion of the study), participants were emailed a follow-up survey. Additionally, within the app, participants answered weekly questions about respite time amounts and respite satisfaction.

### Recruitment Capability

**Figure 2.**
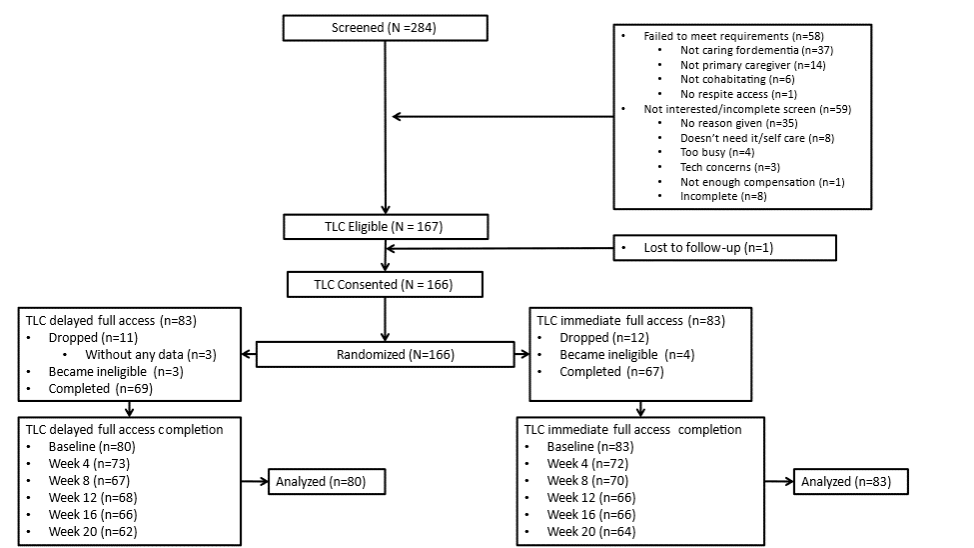
CONSORT diagram of TLC (Time for Living and Caring) immediate (all feature access) and delayed (initial partial access) randomization arms.

Feedback on the TLC intervention and TLC study protocols was sought from a community advisory board and collected via focus groups [24] before participant recruitment. These relationships provided collaborative opportunities to develop recruitment strategies. Collaborations with community groups and health care professionals, and leveraging online platforms, optimized recruitment efforts of the TLC study [40]. Recruitment of eligible caregivers (n=163) occurred from October 2020 to March 2022 (see Figure 2). The TLC sample included dementia caregivers who were, on average, 61.7 years of age (standard deviation 13.0, min 20, max 92). They were primarily spouses or partners (68.1%) or adult children of care recipients (24.1%). Most were female (78.9%), White (82.5%), non-Hispanic (90.4%), married or living with a partner (83.7%), had a college education (89.7%), and had incomes greater than $50,000 annually (73.6%). The participants recruited for the TLC study were generally comparable to national caregiving populations, with identifiable differences that would be expected based on disease focus and geographic focus (eg, age, ethnicity, caregiver, and recipient age) (see Multimedia Appendix 1).

### Practicality

Considering “practicality” as a dimension of feasibility helps researchers make informed decisions about whether the study can be realistically executed within the available resources, time, and logistical constraints. The timeline for the intervention pilot was 5 years (2018‐2023). This included development, recruitment, analysis, and dissemination. Table 2 describes the resources and logistics needed to conduct the TLC pilot study. Per enrollee, it cost US$205 and an average of 12.6 hours of research team time. Access to and willingness to use technology were potential barriers; however, 96% of enrolled participants had an internet-capable device, and 100% had access to the internet. For the pilot study, the intervention was designed for use with desktops, laptops, or tablets, but not for smartphones. Overall, 72% of enrolled participants (117 of 163) preferred the capable devices offered, though the remaining 46 of 163 (28%) preferred smartphone delivery if and when that technology may be available for TLC intervention.

### Adaptability

Assessing the sub-domain of “Adaptability” involves empirically observing and documenting the study’s capacity to flexibly respond to the diverse needs of participants within the scope of the intervention design. We observed whether participants had different experiences by how familiar they were with technology (see Table 2). The pilot study participants had Computer Proficiency Questionnaire scores [41] that ranged from 35 to 60 (the full range of scores could have been 12 to 60), and an average of 56. Though the sample’s average score was high, not all participants were confident using technology and computers. As one participant noted, “It was relatively easy. I’m not very computer savvy, and experienced few problems.” Additionally, the intervention included a button where participants could request support or ask questions when they needed technical support; personal support was then provided by email, phone call, or text.

TLC was also adaptable in terms of how and when the participant could engage with it. By creating TLC as an online web-based intervention, participants could access it when it was convenient for them within the weekly schedule. Additionally, participants could log in as many or as few times as desired and for the duration they needed. Over the 16 weeks of active use, the number of participant sessions ranged from 0 to 67, with an average of 20 sessions per participant. The duration of sessions ranged from 1 to 30 minutes, with the average session lasting 8.5 minutes.

### Social Validity

Social validity refers to the extent to which the intervention is considered meaningful, relevant, acceptable, and effective by the individuals involved; it concerns the social significance or relevance of intervention goals and is traditionally conceptualized as acceptability [26]. Acceptability is a core concept in digital health in part due to its ability to predict and explain key outcomes of interest [42]. Table 3 describes both participants’ and health care providers’ feedback on the specific intervention features and potential future uses of the intervention. Participants and providers were generally close in their assessment of how important or useful the features of the intervention were. One exception was the “resources” feature. Overall, 67% (89 of 132 participants who responded to this item) rated the resources feature as useful; however, this includes the 28% (37 of 132) who reported not using this feature. For those participants who used this feature, 94% (89 of 95) rated the resource feature as useful.

### Generalizability

Generalizability assesses the extent to which the intervention procedures generalize to non-treatment settings, over time, and with diverse samples. While the TLC study was limited to dementia caregivers, the intervention was developed with an eye toward any caregiver. Respite providers were asked, “Do you think that the TLC app could be used with all types of caregivers?” As shown in Table 3, overwhelmingly, 55 of 57 providers answered that the TLC intervention could, or possibly could, be used for all types of caregivers (75% yes, 21% maybe).

Table 3 shows that 127 of 163 (78%) participants would consider using the TLC website in the future. Reasons for future use varied, and in fact, many participants pointed to the variety of uses they got from the app, as shown by one participant who stated, “I like to have a framework to help me refocus from what my daily routine is. I like the resources section. I feel like it would be nice to go back in and once again review those resources. With time, some types of respite change and new ideas are welcomed.” Others mentioned the usefulness of the routine or the “Discipline in keeping track of set goals for caregiver on a daily basis.” Another participant noted, “Again, more than the actual schedule (because I tend to use a paper calendar and daily written to-do lists), the reminder each week to take time for personal self-care was really valuable. It kept me on track. I’m pretty ADD and routines are hard, so the accountability of the website kept me constantly reminded and responsible.”

Often, for those who did not think they would use TLC in the future, the initial intervention was seen as having accomplished its goals or was beneficial in creating awareness of the need for specific goals. One participant noted, “It has already helped me plan ahead and think of what I will be doing. I don’t think I need that assistance anymore but stand to be corrected!” This indicates that future use would be possible if they felt they had stopped planning and taking time for respite. Others may have taken some of the intervention goals, such as being specific, and incorporated them into other systems - “I have an online calendar where everything in my life (and my mom’s) is tracked - this would duplicate that effort. BUT what I did like about the site was that I was specific as to what the respite was scheduled for - I don’t have that level of detail on my calendar.*”*

### Integration

Assessing integration concerns how aligned the intervention is with existing practice and infrastructure. Table 3 shows that 148 of 163 participants and 51 of 57 providers (~90% of each group) would recommend the TLC app to caregivers. However, the method of delivery may be individually and situationally based. In total, 78% (103/132) indicated that they would use TLC optimized for a smartphone. Providers were asked how they preferred to deliver support to caregivers, including as an app or interactive website, workbooks, or worksheets (fillable, printable, pencil and paper), in-person personal coaching, or personal coaching using telephone or video conference. A self-guided app or interactive website and in-person personal coaching tied for first, with 17 of 57 providers (30%) ranking each as first choice. Table 4 provides exemplars of why participants would or would not use the TLC app optimized for a smartphone. While most participants would use TLC as a smartphone app, which would cover those who have it as a preferred method, the ability to use different preferred devices is preferable overall.

### Implementation

As noted earlier, providers indicated that they would be willing to recommend the intervention to current and future respite clients, but another critical question is whether the intervention could be implemented by providers in the same manner as the research team. As an online web-based intervention, TLC was implemented with high fidelity (eg, as intended by the developers and researchers; the same messaging and imagery were delivered to participants based on the algorithms underlying the software). A small fraction of participants (<10%) accessed technological support from TLC research staff; this kind of human interaction and support would likely not be available if the app were implemented outside of the research study environment.

### Effectiveness

Table 5 describes the metrics we used to identify whether the TLC intervention brought about positive change for the participants. Participants reported an increase in the amount of respite received each week, from an average of 8 hours at the start of the intervention to an average of 13 hours at the end of the intervention. The percentage of participants who felt they were getting enough respite time increased from 10% to 42% (16 participants to 68 participants). Participants were more likely to schedule their respite hours (58% vs 90%) and were happier with what they did during respite (72 or 44% vs 119 or 73%) after the intervention, compared to before. As noted by a participant, “TLC helps me focus on what I want to do with my time. It helps me plan to do things that really nurture me.” There were no differences in participants’ average depression or burden scores, but there was a slight increase in their perception of positive aspects of caregiving. Additionally, the average scores do not preclude individual benefits that may have been gained by some, as noted by a participant who stated, “Having a goal of respite each week has been helpful and has reduced some of my anxiety.*”*

**Table 5.** Measures of intervention effectiveness.

Measure^a^	Pre-intervention	Post-intervention
Caregiver and respite outcomes		
Amount of respite per week (in *hours*), mean (SD)	8 (11.5)	13 (11.7)
I am getting enough respite time, %	10	42
My respite is scheduled in advance, %	58	90
I am happy with what I did during respite, %	44	73
Depression (T-score scale range 37.1‐81.1), mean (SD) [39]	54.1 (7.4)	53.6 (7.8)
Burden (scale range 0‐96), mean (SD) [43]	46 (16.6)	45.9 (17)
Positive Aspects of Caring (scale range 9‐45), mean (SD) [44]	25.5 (9.7)	28.4 (9.4)
Perceived benefits of intervention		
Using the TLC intervention was a positive experience for me, %	—	85
TLC intervention helped me improve my respite time to moderate*, %*	—	83
I feel happier, healthier, or less stressed after using TLC intervention, *%*	—	92

a All measures were self-reported by caregivers who had used the TLC intervention. The sample used to calculate descriptive statistics for this table was restricted to those who had data at both pre- and post- assessment (depression n=163; burden n=155; positive aspects n=159; perceived benefits n=132).

Overall, of 132 participants who responded to perceived benefits items, 112 (85%) rated their experience using the TLC intervention as positive or very positive. Only 1% (2 of 132) reported a negative experience, while 14% (18 of 132) felt neutral. Participants reported being able to identify times that they may not have considered before. As one participant stated, “I am learning that scheduling respite ahead of time is very helpful. I am actually able to schedule more time than I realized and still give the care needed – we are both happier.” Another participant noted that they were able to focus on using the time available, “Respite becomes more valuable when the time and activities are scheduled. Even if the activity is to disappear and do nothing. Without a respite schedule, the time gets squandered.” The TLC intervention also helped provide validation for some. As one participant stated, “Budgeting time to myself is paramount to being a good caregiver. Seeing the planned times on the calendar gives me value as a person of worth.” Another participant stated, “Honestly, I am taken back about how just calendaring time out has made such a difference – thank you!”

At the end of the 16-week intervention period, a majority of participants (134/159, or 83%) reported that they felt the intervention improved their respite time use, and more than 9 out of 10 participants (92%) reported that using the intervention made them happier, healthier, or less stressed. As one participant stated, “I like that I schedule my respite. I like that the time I do have to myself or the time I scheduled is labeled respite. It makes me appreciate that I really do have carved out time to stop the world and focus on other things I love to do.” The intervention process was noted by another who found, “The process of scheduling time off (and making arrangements to be able to take time off) has been a real boon!*”*

## Discussion

### Principal Results

Using a comprehensive mixed-methods approach to feasibility analysis [38], we used pilot data from the TLC study to evaluate a respite-focused caregiver intervention in terms of 10 conceptually distinct measures of feasibility, as outlined by Gadke et al [26] (see Table 1 for definitions of feasibility). Overall, across each domain, we found strong indicators and evidence of feasibility for the TLC intervention. Thus, we conclude that the TLC intervention is feasible. This feedback also provides a glimpse into the potential or promise of the intervention in supporting family caregivers in their use of respite. Providing caregivers with coaching and resources to recognize the value of respite and to maximize the benefit of whatever respite time they have by using goal-setting and goal-review techniques has the potential to provide meaningful support for family caregivers. This respite-focused intervention may be particularly relevant for respite service providers to ensure that their clients are receiving maximum benefit from their respite services (which oftentimes have long waitlists meaning that respite services are fairly limited), as well as for policy makers and caregiver advocates who may need further empirical evidence showing the benefits of respite to advocate for increased funding for respite.

This analysis also provides a case study that can serve as a model for other researchers who may be developing interventions and the study designs that will be used to evaluate the feasibility of the new intervention. Adopting such a comprehensive approach to feasibility assessment allowed us to design a pilot study that included multiple measures of feasibility, rather than only an isolated set of measures tacked on as an afterthought to a study that focused primarily on the measurement of primary outcomes. For example, we decided to use a fully powered sample for this pilot study because it would allow us to get a better sense of initial efficacy and identification of mechanisms that underlie the intervention’s effect. Additionally, by choosing to work with a community advisory board representing the diverse caregiving experiences and multiple cultural perspectives, we were able to make significant refinements during the initial TLC technology build, rather than having to wait until the pilot study was completed to learn about what those refinements should be. Doing a systematic and iterative development process where we co-created the intervention with a community advisory board did cost money and took time, but it provided richer data and a more refined TLC intervention for the pilot study than would have been available without that initial investment and commitment to developing a feasible product from the start.

Pilot studies, although focused primarily on issues of feasibility, can and should be as comprehensive as possible so that we can develop interventions that have the potential to be implemented beyond small-sample pilot studies. By using the Gadke et al [26] 10-dimension perspective, we developed a rich and comprehensive set of data collection instruments and metrics that we could use to assess the complexity of feasibility. For example, in response to the “generalizability” dimension of feasibility, we decided to include a separate data collection effort with a nationwide sample of respite providers. While this additional step added time and effort to the TLC study, it provided us with information and insights about the possible implementation of TLC. Relatedly, intervention research should also engage in cost analyses, assessing whether the benefits of an intervention have measurable cost savings. We assessed how much the intervention cost us to build but have not yet assessed whether using the intervention saved participants or providers money. Future research should include methodology and metrics to assess the cost-effectiveness of interventions [45].

While there are no costs associated with the delivery of the intervention once the web platform is built, there are significant costs associated with the initial build, as well as the refinements that are needed at each stage of evaluation and overall maintenance and general updates and bug fixes. Navigating through the stage-model for intervention development [21]—where each stage may take several years to complete—may be too slow to create an effective intervention, especially for technology-based interventions where the style and functionality change very quickly with the rapid introduction of new apps and features available to the consumer or public. This consideration may help explain the differences for those who reported a neutral experience using TLC, but also reported *any* improvement (from slightly to extremely) in their feeling happier, healthier, or less stressed as a caregiver after using TLC. The style and functionality may have impacted their experience, even though they were able to still see some benefit from the intervention.

### Conclusions

Overall, these results suggest that TLC should be further refined and move toward later stages of intervention research, where the most essential features of the intervention can be implemented in less controlled research environments. Based on these results, combined with findings that TLC improves caregiver well-being [34], TLC is likely ready for a large-scale implementation trial “to test real-world transferability and effectiveness” (p. 33) [20]. Moving toward this goal, the key features of the TLC intervention have been made available on a publicly accessible webpage for caregivers to access and use for free [35]. A major goal of this feasibility analysis was to assess whether a fully self-administered, web-based intervention is feasible for family caregivers, who tend to be among older generations that have less computer proficiency and access to internet and internet-capable devices [20]. The TLC pilot study found that there is certainly a subgroup of older caregivers who are responsive to and seeking this kind of support. This helps us shed the outdated tropes that older adults do not want to access support and resources online [46-48].

## Supplementary material

10.2196/71792 Multimedia Appendix 1 Comparison of TLC sample to national profile of family caregivers.

## Abbreviations

TLC: Time for Living and Caring
