# Supplementary material for: Assessing the Time for Living and Caring (TLC) Study: Mixed-Methods Feasibility Study of a Web-Based Caregiver Intervention to Improve Respite
Source: JMIR Aging. 2025 Aug 18;8:e71792. doi: 10.2196/71792 (PMC12360673; doi:10.2196/71792)
Supplement: Multimedia Appendix 1 [file aging-v8-e71792-s001.docx]

**Multimedia Appendix 1: Comparison of TLC sample to national profile of family caregivers**

|  | **TLC Sample**  (n = 163) | **2020 NAC/AARP Sample^a^**  (n = 1,392) |
| --- | --- | --- |
|  | *Dementia Caregivers* | *Caregiver to Any Adult* |
| **Caregiver Gender** |  |  |
| Female | 78% | 61% |
| Male | 22% | 39% |
|  |  |  |
| **Age of Caregiver** |  |  |
| 18-34 | 4% | 24% |
| 35-49 | 11% | 23% |
| 50-64 | 43% | 35% |
| 65-74 | 26% | 12% |
| 75+ | 16% | 7% |
| *Mean age* | *61.6* | *49.4* |
|  |  |  |
| **Race/Ethnicity of Caregiver** |  |  |
| White | 83% | 61% |
| African American | 6% | 14% |
| Hispanic | 6% | 17% |
| Asian American | 2% | 5% |
| Other | 3% | 3% |
|  |  |  |
| **Caregiver Education** |  |  |
| Less than college degree | 44% | 65% |
| College graduate or higher | 56% | 35% |
|  |  |  |
| **Caregiver Marital Status** |  |  |
| Married | 84% | 54% |
|  |  |  |
| **Caregiver Household Income** |  |  |
| Less than $50,000 | 25% | 36% |
| $50,000 or more | 75% | 64% |
|  |  |  |
| **Caregiver Living Location** |  |  |
| Urban/Suburban | 85% | 88% |
| Rural | 15% | 12% |
|  |  |  |
| **Caregiver Employment Status** |  |  |
| Employed while caregiving | 38% | 61% |
| Not employed | 62% | 39% |
| **Care Recipient Age** |  |  |
| Age 50+ | 97% | 87% |
|  |  |  |
| **Living Situation** |  |  |
| Co-residence with caregiver | 100% | 40% |
|  |  |  |
| **^a^**AARP/NAC Caregiving in the U.S. 2020 Report^1^ | | |
